# Formulation, Optimization and Evaluation of Cytarabine-Loaded Iron Oxide Nanoparticles: From In Vitro to In Vivo Evaluation of Anticancer Activity

**DOI:** 10.3390/nano13010175

**Published:** 2022-12-30

**Authors:** Ritesh Fule, Mohammed Kaleem, Turky Omar Asar, Md Abdur Rashid, Rasheed A. Shaik, Basma G. Eid, Mohammed Z. Nasrullah, Aftab Ahmad, Imran Kazmi

**Affiliations:** 1 Department of Pharmaceutics, Dadasaheb Balpande College of Pharmacy, Besa, Nagpur 440036, Maharashtra, India; 2 Department of Pharmacology, Dadasaheb Balpande College of Pharmacy, Besa, Nagpur 440036, Maharashtra, India; 3 Department of Biochemistry, Faculty of Science, King Abdulaziz University, Jeddah 21589, Saudi Arabia; 4 Department of Biology, College of Science and Arts at Alkamil, University of Jeddah, Jeddah 23218, Saudi Arabia; 5 Department of Pharmaceutics, College of Pharmacy, King Khalid University, Al Faraa, Abha 62529, Saudi Arabia; 6 Department of Pharmacology and Toxicology, Faculty of Pharmacy, King Abdulaziz University, Jeddah 21589, Saudi Arabia; 7 Health Information Technology Department, Faculty of Applied Studies, King Abdulaziz University, Jeddah 21589, Saudi Arabia; 8 Pharmacovigilance and Medication Safety Unit, Center of Research Excellence for Drug Research and Pharmaceutical Industries, King Abdulaziz University, Jeddah 22252, Saudi Arabia

**Keywords:** trehalose, cytarabine, iron oxide nanoparticle, bioavailability studies, cell line studies

## Abstract

Innovative drug delivery systems based on iron oxide nanoparticles (INPs) has generated a lot of interest worldwide and have prime biomedical benefits in anticancer therapy. There are still issues reported regarding the stability, absorption, and toxicity of iron oxide nanoparticles (INPs) when administered due to its rapid surface oxidation and agglomeration with blood proteins. To solve this problem, we have synthesized trehalose-coated stabilized iron oxide nanoparticles (TINPs) by a co-precipitation technique. The surface coating of INPs with trehalose helps to improve the stability, prevents protein binding, and increase absorption uptake inside the body. Developed TINPs was then loaded with anticancer drug cytarabine by chemical crosslinking encapsulation method using suitable solvent. Engineered cytarabine-loaded trehalose-coated stabilized iron oxide nanoparticles (CY-TINPs) were optimized for particle size, zeta potential (−13.03 mV), and solid-state characterization such as differential scanning calorimetry (DSC), X-ray powder diffraction (XRD), and transmission electron microscope (TEM) studies. The particle size of 50 nm was achieved for developed CY-TINPs. The developed CY-TINPs was further evaluated for in vitro cell line investigations which confirmed potential cytotoxic activity. Developed CY-TINPs show remarkable enhancement in in vivo pharmacokinetic parameters C_max_ as 425.26 ± 2.11 and AUC_0–72_ as 11,546.64 ± 139.82 as compared to pure drug. Compared to traditional drug delivery, the CY-TINPs formulation can effectively delay release, improve bioavailability, and boost cytotoxic activity against tumors.

## 1. Introduction

Cancer is a group of complex diseases that arise from aberrational cell growth due to deregulated division of cells and it eventually may metastasize to other organs of the body [1,2]. Globally, cancer is the one of the major cause of mortality among all the diseases [3]. This disorder is preceded by cancer cell proliferation, differentiation, migration, and apoptosis abnormalities [4]. Even though popular therapeutic materials are used to treat cancer and other diseases, nanostructured materials (INPs) have become among the key biomaterials for diagnosing and treating intricate disorders including various contagious diseases and cancers [5]. Tissue engineering has expanded the spectrum of anticancer therapy as well as offered better compliance to patients [6].

Cytarabine, doxorubicin, paclitaxel, etc., are used for cancer treatment. Cytarabine, a cyclic pyrimidine nucleoside, inhibits cell division by crosslinking DNA. This drug tackles retinoblastoma, neuroblastoma, lung cancer, and breast cancer [7]. Cytarabine is effective in treating nonlymphocytic leukemia, and its activity is limited to the S phase [8]. To obtain optimum cytotoxic activity, cells must be exposed to cytotoxic concentrations over an extended period. Cytarabine is quickly broken down into a metabolite called uracil arabinoside [9], which is not active in the body. Oral administration of cytarabine is not possible because it degrades in body fluids and liver bypass within an hour. IV formulation is the only available choice for long-term administration, which has toxicity issues. Thus, using an external magnetic field to target therapeutic candidates at the tumor site could ensure a high concentration of drug and minimal adverse effects.

Nanomedicine is a new field that uses knowledge and techniques from nanoscience to help with the prevention and treatment of disease. The first generation of FDA-approved nanoparticle-based therapies consists of lipid systems such as liposomes and micelles [10]. FDA-approved formulation products such as liposomes and micelles contain inorganic nanoparticles including silver, INPs, and gold [11]. Iron oxide nanoparticles are a biocompatible nanoscale material that can achieve cellular bioactivity in body fluids. INPs can be tagged with low-molecular-weight peptides, polymers, microbial enzymes, and plant proteins to prevent interaction with blood serum proteins [12]. This functionalization of INPs surface using polymeric excipients increase the stability of INPs in blood and it subsequently increase its blood circulation time.

Nanoparticles’ ability to produce oxidative stress is largely responsible for their harmful effects on cells and organisms. INP-induced iron degradation causes non-oxidative stress [13]. Through the reduction reaction, this process forms peroxy free radicals and hydroxy from hydroxyl radicals. An excess of reactive oxygen substances can alter protein and gene expression, cause peroxidation, and damage DNA (ROS) [14]. Apoptosis and necrosis are also possible outcomes of oxidative stress on cell signaling cascades [15]. Cellular carcinogenicity is another possible outcome. To avoid these problems, researchers developed functionalized INPs with biocompatible polymeric coatings derived from polyacrylic acid for INP stabilization [16]. Lin and colleagues [17] coated INPs with polymeric polyethylene glycol-block-allyl anhydride ether (PEG-b-AGE) and showed that under an external magnetic field, coated INPs separated specific tumor cells from an aqueous environment. Another scientist reported that PEG/PEI (polyetylene imine) and PEG (polyethylene glycol) was used as coating materials for surface functionalization of INPs [18]. Panda and colleagues [19] created synthetic polymeric INPs and used them to deliver docetaxel to breast cancer cells in a targeted manner. Due to their morphological features, INPs can form colonies in cell membranes. So, drug-loaded nanoparticles with polymeric coating accumulate at target region, increasing drug concentration and minimizing toxicity. However, biomimetic surface coatings can reduce or eliminate the risks associated with metal and metal oxide nanoparticles [20].

Understanding the molecular and toxicity aspects of INPs, we have engineered smart nano drug delivery system combining nano precipitation and impregnation technique. The objective of this research is (I) engineering stable prolonged release stable CY-TINPs nano formulation, its optimization and solid-state characterization, (II) physicochemical characterization of solid state, (III) evaluate chemopreventive effects of drug-loaded nanoparticles on human cancer cell lines, and (IV) in vivo pharmacokinetic bioavailability study. Our prime goal is to overcome problems associated conventional cytarabine dosage systems and improve patient compliance for anticancer therapy using CY-TINPs. Developed CY-TINPs formulation undoubtedly will be more advantageous than previously reported drug delivery systems.

## 2. Materials and Methods

### 2.1. Materials

Cytarabine drug was generously gifted by Cipla Pvt. Limited, Mumbai, India. FeCl_3_. 4H_2_O, FeCl_3_, (employed for engineering iron oxide), adriamycin, DMSO was obtained from Sigma Aldrich. The cell lines MTT kit (3-(4,5-dimethylthiazol-2-yl)-2,5-diphenyl tetrazolium bromide), Ficoll-HiSep, foetal bovine serum (FBS), trypan blue, penicillin, streptomycin was procured from Loba Chemie Pvt ltd, Mumbai, India. Trehalose was procured from Mitushi biopharma, Ahmadabad, Gujrat. All the solvents such as methanol, acetone, and acetonitrile were of HPLC grade procured from SD Fine Chemicals Pvt. Ltd., Mumbai, India. HPLC grade water was used in all the experimental procedures.

### 2.2. Formulation Methodology

#### 2.2.1. Synthesis of Trehalose Coated Iron Oxide Nanoparticles (TINPs)

Iron oxide nanoparticles were synthesized by an alkaline co-precipitation method from heating an aqueous solution of FeCl3 and FeCl2.4H2O (2:1) in a nitrogen atmosphere at 80 °C followed by precipitation with 3 M NaOH as reported in the literature [20,21]. In a beaker, solution of water and ethanol in a ratio of 7:3 was prepared. Into this solution, 3% trehalose was added drop wise with magnetic stirring at 50 rpm constant speed. Trehalose molecules were adsorbed on the surface of INPs and so called termed as trehalose-coated INPs (TINPs). After 20 min of process, black precipitate was formed and collected. This obtained raw material was then washed with double distilled water and lyophilized to get trehalose-coated iron oxide nanoparticles (TINPs). The TINPs were analyzed for its particle geometry and other solid phase identification properties were quantified using available analytical tools.

#### 2.2.2. Formulation of Cytarabine Loaded TINPs (CY-TINPs)

First, 10 mg cytarabine in powder form was dissolved in 30 mL of acetone and then 30 mg of TINPs were added to it. To this solution, 1 mL of PEG (2% *v*/*v*) solution was added drop wise with continuous stirring. PEG acts as a crosslinking agent for the loading and encapsulation of drug inside TINPs. The solution mixture was stirred for 3 h. and till 24 h acetone evaporated at room conditions, and the CY-TINPs powder formed was collected. Obtained CY-TINPS was freeze dried and further evaluated for drug content and physical characterization.

#### 2.2.3. Drug Content Analysis by HPLC

The HPLC assessment of cytarabine was performed with slight modifications to the previously reported method [22]. HPLC grade water was taken and made to pH 2.8 by adding approximate amount of orthophosphoric acid (aqueous phase). Mobile phase containing acetonitrile and prepared above aqueous phase (having pH 2.8) was prepared in a ratio of (2:98 *v*/*v*) respectively. Above mobile phase was delivered at a flow rate of 1 mL/min in HPLC C18 Column (ZORBAX Eclipse Plus, 5 Å, 5 µm, 4, 6 × 250 mm by Agilent Corporation, Milford, MA, USA) at 280 nm wavelength. At 6.8 min, the drug quantifying peak was detected. Linearity range was obtained at concentrations between 50 and 200 ppm.

### 2.3. Physicochemical Characterization

#### 2.3.1. FT-IR Analysis

A Shimadzu FTIR spectrometer was used to study the FTIR spectrum (IR Affinity 1Model, Kyoto, Japan). Cytarabine, TINPs, and cytarabine-loaded TINPS were tested and scanning range was set between 4000 and 500 cm^−1^.

#### 2.3.2. Differential Scanning Calorimetry

Differential scanning calorimeter (DSC-PYRIS-1, Perkin Elmer, MA, USA) was used to study the thermal behavior of pure drug and developed formulation. The samples were heated at a rate of 10 °C min^−1^ from ambient temperature to above drug melting point till 300 °C. Cytarabine, TINPs, and cytarabine-loaded TINPS samples (5.0–10.0 mg) were carefully weighed into sealed aluminum pans. These DCS pans were heated at 10 °C/min under a nitrogen purge (20 mL/min) from 20 °C to 300 °C. An empty crimped aluminum pan was used as the reference cell. The DSC was calibrated for baseline using empty cells and for temperature [23].

#### 2.3.3. Powder X-ray Diffractometry (PXRD)

The solid phase of cytarabine, trehalose, plain TINPs, and cytarabine-loaded TINPs (CY-TINPs) was investigated using an X-ray diffractometer. XRD instrument used is Bruker D8 Advance, (Wisconsin, USA). Samples about 100 mg was placed under the chamber where beam of rays scan with speed of 2°/min over a range of 5–60 (2u). This system have improved sensitivity for lowered concentration of sample, facilitating for quantitative study of micro crystalline and amorphous materials [24].

#### 2.3.4. Particle Size and Zeta Potential Analysis

Photon correlation spectroscopy (PCS), which studies variations in light scattering caused by Brownian motion of particles, was used to assess the droplet size distribution and polydispersity index (PDI) of the formulation. The particle sizes and zeta potential of the CY-TINPs were measured using a Zetasizer Nano-ZS90 (Malvern Instruments, Malvern, UK). The detection range was between 0.8 nm and 6.5 m. To measure, 0.1 mL of the formulation was added to 100 mL of water and the flask was gently inverted 4–5 times. Then a few milliliters of aliquot are taken and placed in the sample cell for droplet size measurement. Light scattering was measured using zeta potential at 25 °C and at 90° angle. At room temperature, measurements were taken in triplicate. CY-TINPs (1 mg/mL) dispersed in distilled water to measure the zeta potential [25].

#### 2.3.5. Transmission Emission Microscopy Analysis

Transmission electron microscopy pictures of nanoparticles were acquired to elucidate their shape and configuration. Transmission electron microscopy (TEM) was used to examine the surface morphologies of the CY-TINPs using a model H-7600 microscopic lens (Hitachi, Tokyo, Japan) [26].

#### 2.3.6. Drug Loading (DL) and Entrapment Efficiency (EE)

The content of cytarabine found in CY-TINPs was determined and investigated. In this technique, 10 mL of 0.1 M HCl was added to 50 mg of newly synthesized CY-TINPs. To separate the unentrapped drug from the solution, the solution was ultra-centrifuged at 15,000 rpm and 4 °C (Cooling Centrifuge Remi, Mumbai, India). The remaining titer of solution, which only contained free drug, was discarded. Suspended CY-TINPs was collected and rinsed sufficiently with distilled water. Obtained solution was then heated in methanol at 80 °C and again diluted with distilled water. The concentration of cytarabine in water was determined using the HPLC technique. The drug loading (DL) and entrapment efficiency (EE) were calculated by using following equation [27,28].

(1) %EE=amt. of cytarabine suspended/amt. of cytarabine added × 100

(2) %DL=amt. of cytarabine suspended/amt. of cytarabine added+amt. of excipients added × 100

#### 2.3.7. In Vitro Drug Release

The developed CY-TINPs formulation target is to prolong and sustained the release and for that drug so that present in blood over an extended period. So, it is important for evaluating its properties and behavior. The release of drug from CY-TINPs was investigated using a dialysis bag method using phosphate buffer of pH 7.4 [29]. Nanoparticles are held by the dialysis bag, which lets drugs with a molecular weight of less than 12 KD run back freely into the dissolution medium. About 2 mL of solution containing CY-TINPs were put into a bag, one end wrapped in thread. The bag was positioned within a beaker containing 100 mL of dissolution medium (PBS pH 7.4) was added. The bag is then placed inside the beaker (hanging with thread) with magnetic stirring 100 rpm speed at room temperature. After 1, 2, 3, 4, 5, 6, 12, and 24 h, followed by up to 120 h at 24 h intervals, aliquots were removed in the conical flask and replaced with new medium, maintaining sink condition. The filtrate was subsequently analyzed by HPLC.

#### 2.3.8. Cell Viability Assay CY-TINPs Formulation

The toxicity of CY-TINPS formulation of 15, 30, 50, and 100 µg/mL was tested by incubating them with normal human lymphocytes. Density gradient centrifugation was used to separate the cells. Freshly collected peripheral blood was used to extract lymphocytes, which were subsequently added to anticoagulant EDTA. Blood was initially diluted in 1:1 ratio with phosphate buffered saline. Then, 10 mL of diluted blood was injected precisely with undisturbed the interface after 3 mL of Ficoll-HiSep had been added to a 15 mL centrifuge. This was followed by a 30-min centrifugation at 1800 rpm. After 10 min, we separated and washed the lymphocyte ring three times with PBS. Cells were cultured in RPMI media with 10% heat-inactivated fetal bovine serum (FBS) supplemented with 100 U/mL penicillin and 100 g/mL streptomycin at 37 °C in a humidified atmosphere of 5% carbon dioxide. The effect of CY-TINPS on mononuclear cells was measured at concentrations of 15, 30, 50, and 100 µg/mL (1 × 10^5^ cells/mL). The culture without formulation was used as negative control and adriamycin (1 µM) as a positive control was used to measure cell growth. After 72 h, 20 mL of 3-(4,5-dimethylthiazol-2-yl)-2,5-diphenyl tetrazolium bromide (MTT) solution of concentration (10 µg/mL) was added to each well. These plates were centrifuged for 25 min at 3000 rpm after being incubated at 37 °C in a CO2 incubator for 4 h. After the formazan crystals had been treated with DMSO, the absorbance of each well was measured at 570 nm using an ELISA reader at 25 °C (100 mL) [30,31].

#### 2.3.9. Anticancer Activity

The Centre for Cellular & Molecular Biology (CCMB), Hyderabad, India, provided the HL60 acute promyelocytic leukemia (APL) line cells for this study. The cells were cultured in RPMI media containing 10% heat-inactivated fetal bovine serum (FBS), 100 U mL^−1^ penicillin, and 100 g^−1^ streptomycin in a humidified environment containing 5% carbon dioxide. The trypan blue method was used to measure cell viability after a 48 h. incubation. After reaching a density of 5 × 10^4^ cells/mL, in vitro cell line investigation was commenced. Cell growth inhibition was used to evaluate the cytotoxic action of cytarabine, blank TINPs and cytarabine-loaded TINPs (CY-TINPs). MTT-based calorimetric assay method was used for the cell growth inhibition investigation. To facilitate exponential growth, 3 × 10^4^ cells per ml of HL60 cell lines were plated in 24-well plates. For 72 h, the cells were exposed to cytarabine (15–100 µg/mL) and cytarabine-loaded TINPs (CY-TINPs) in a controlled situation. Then, a 20 µL MTT solution (10 mg/mL concentration) was added to each well, and the plates were incubated at 37 °C for 4 h to allow the MTT to be reduced by living cells, resulting in the formation of purple formazan crystals. Each well’s absorbance was measured at 570 nm using an ELISA reader at 25 ± 1 °C after the formazan crystals were dissolved in DMSO (100 mL). An inverted phase contrast microscope was also used to examine the cellular structure [31,32].

### 2.4. Experimental Animal Studies

To conduct this study, albino (wistar) rats were purchased from Department of Laboratory Animal veterinary sciences, Amravati. Each experiment was approved by the Institute’s animal ethical committee and conducted in compliance with CPCSEA guidelines. The animals were treated with compassion and empathy and were never harmed or stressed in any way. The animals were housed in individual anodized cages in groups of 5 with their respective air conditioning systems. The animals were kept in an environment with a constant temperature (22 °C), humidity (30–70%), and a light/dark cycle of 12 h per day. Throughout the course of the experiment, adequate food and water were provided to the animals. For the in vivo studies, albino rats weighing between 160 and 220 g were employed.

#### 2.4.1. In Vivo Pharmacokinetic Studies

Planned procedures for sampling were proposed to study in vivo performance of the prepared cytarabine-loaded TINPs (CY-TINPS). The bioavailability of a single dose was investigated in fasted Wistar male rats. Bioavailability studies were carried out in male wistar rats using a dosage of 10 mg/kg body weight to determine oral bioavailability for both the improved CY-TINPs and the cytarabine drug solution. Animal experimental protocols were examined and authorized by our institute’s animal ethics committee. (IAEC/AMT-012022-E4). Wistar male rats weighing between 200 and 250 g were used in this investigation (each group contained 06 animals). Puncturing the retro-orbital venous plexus yielded blood samples at 0 (pre-dose), 0.5 (post-dose), 1, 2, 3, 4, 6, 8, 24, 32, 40, 48, 56, 64, and 72 h. At each time point, 0.5 mL of blood was taken in an Eppendorf, after which they were swirled at 3000 rpm for 30 min in a centrifuge. The serum was thereafter placed in a different eppendorf tube and refrigerated at 20 °Cs until analysis.

#### 2.4.2. Determination of Cytarabine in Rat Plasma

Cytarabine was separated using HPLC by retaining the column at a specific temperature of 40 °C. HPLC Column (ZORBAX Eclipse Plus, C18, 95 Å, 5 µm, 4, 6 × 250 mm by Agilent Corporation, Milford, MA, USA) was used for the analysis. Drug quantification in blood plasma was determined at 280 nm wavelength at a flow rate of 0.5 mL/min. Gradient elution method was implemented containing mobile phase A of 0.1% (*v*/*v*) formic acid aqueous solution and a mobile phase B of 100% acetonitrile (2:98 *v*/*v* ratio). The drug peak was identified at 8.5 min [33].

#### 2.4.3. Statistical Investigation

For quantitative analysis of the collected data, analytical applications such as Graph Pad prism and Excel sheets were utilized. The data were presented as means with standard deviations. To assess statistical differences, a one-way analysis of variance was utilized (ANOVA). P0.05 was considered statistically significant. The graphs were displayed with their standard error bars.

## 3. Results and Discussion

**Figure 1:**
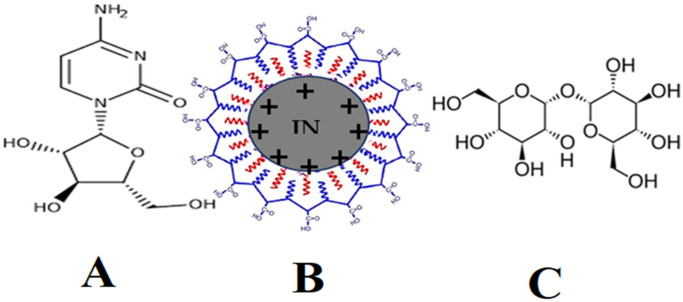
Shows the chemical structure of cytarabine (**A**), iron oxide nanoparticles (**B**), and trehalose (**C**).

Iron oxide nanoparticles, like other nanoparticles, are intrinsically unstable and prone to agglomeration [34]. INPs have large surface area due to nanometer geometry and have a tendency to form hydrophobic interactions and generating bigger clusters as shown in Figure 1 and INPs are readily engulfed by the immune system and thus prevented from reaching their intended target site [35]. Furthermore, they are extensively metabolized in the body fluids, resulting in a loss of functional and magnetic properties, and rendering them inefficient.

Polymeric coating on the surface of INPs provides stability to the nanoparticles, with the goal of reducing the toxic effects [36]. Polymer-functionalized INPs have attracted a great deal of importance in applied research. Due to the polymeric coating, the nanoparticles increase the repulsive forces to counterbalance the van der Waals attractive forces functional on the nanoparticles [37]. However, the saturation magnetization functionality of INPs decreased to some extent due to the coating’s effect on particle size. While the nanoparticle’s bio-distribution profile has improved, its clearance and tissue penetration remain low. Polymers such as trehalose, arabinogalactan, starch, glycosaminoglycan, styrene, polyethylene glycol (PEG), and polyvinyl alcohol are frequently employed for functionalization INPs [38,39]. Trehalose is a polysaccharide that is used to functionalize iron nanoparticles due to its biocompatibility. It has been found that the hydroxyl group present in trehalose chains forms hydrogen bonds with the iron oxide particle surface, which is responsible for the physical adsorption onto the surface of the iron oxide core [40,41]. The adsorption is boosted because the high concentration of hydroxyl groups in trehalose chains greatly enhances the total bonding energy of hydrogen bonds. Trehalose that has been attached to a surface is biomimetic because it resembles the bonding pattern between drug structures and INPs. Trehalose is resistant to in vivo enzymatic degradation.

### 3.1. Physicochemical Characterization

#### 3.1.1. Spectroscopic Studies Using IR

**Figure 2:**
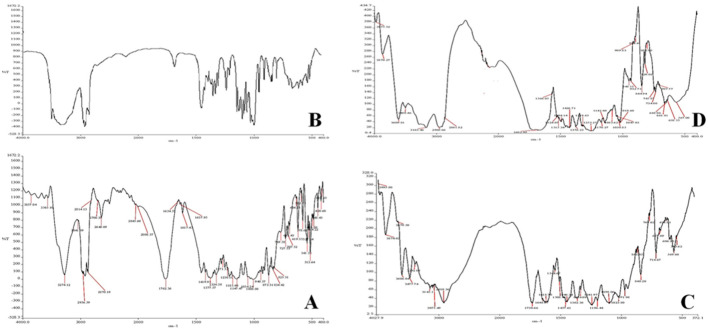
FT-IR Spectra of pure cytarabin (**A**), trehalose (**B**), cytarabine-loaded TINPs (CY-TINPs) (**C**), and CY-TINPs (**D**) after 3 months.

The potential interaction between the drug, trehalose, and CY-TINPs was investigated using FTIR spectroscopy. The main FTIR peaks of pure cytarabine, TINPs, and CY-TINPs interpreted. There was no significant change in the location of the drug’s typical absorption bands and bonds of different functional groups. The FTIR spectra data showed in Figure 2A–C that there was clear drug–TINPs interaction.

#### 3.1.2. Spectroscopic Studies Using DSC

**Figure 3:**
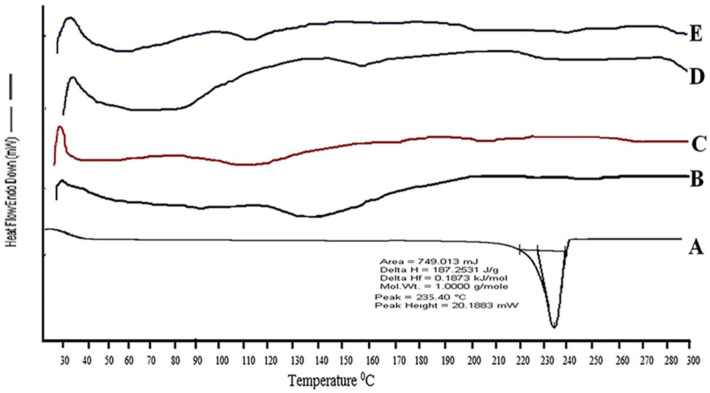
DSC spectra of pure cytarabine (**A**), trehalose (**B**), neat TINPs (**C**), cytarabine-loaded TINPs (CY-TINPs) (**D**), and CY-TINPs (**E**) after 3 months.

DSC analysis was used to examine the thermotropic behavior and physical state of the CY-TINPs. Figure 3A–D depicts the DSC thermograms of cytarabine, trehalose, TINPs, and CY-TINPs. The phase transition peak for pure drug at 232.72 °C with considerable enthalpy confirmed the cytarabine crystalline nature (Figure 3A). The absence of a specific cytarabine peak in CY-TINPs formulation after 3-month stability indicated that the drug’s physical state is stable (Figure 3E).

#### 3.1.3. Spectroscopic Studies Using XRD

**Figure 4:**
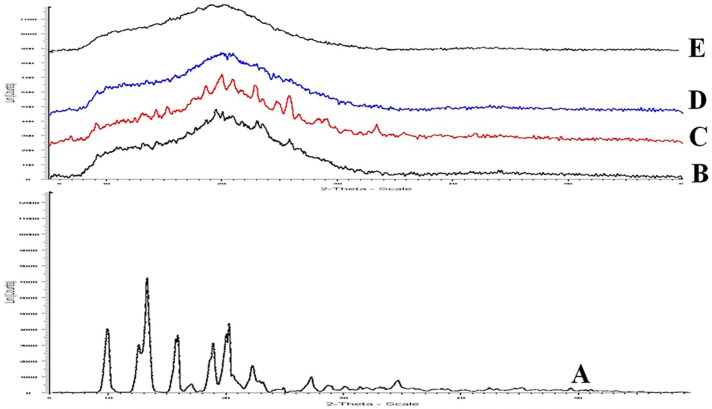
XRD Spectra of pure cytarabine (**A**), trehalose (**B**), neat TINPs (**C**), cytarabine-loaded TINPs (CY-TINPs) (**D**), and CY-TINPs (**E**) after 3 months.

Figure 4 shows a PXRD spectrum for cytarabine drug, trehalose, and CY-TINPs XRD pattern overlay. The pure drug exhibited many high intensity diffraction peaks at 7.41, 9.85, 10.55, 11.23, 12.33, 13.55, 21.20, 22.13, 24.15, and 31.24, showing the substance’s crystalline composition (Figure 4A). XRD pattern of trehalose and neat TINPs shown in Figure 4B,C. The absence of cytarabine distinctive peaks in CY-TINPs formulation (Figure 4D) indicated drug encapsulation inside the TINPs core and stability sample XRD pattern as shown is Figure 4E.

#### 3.1.4. Particle Size and Zeta Potential

**Figure 5:**
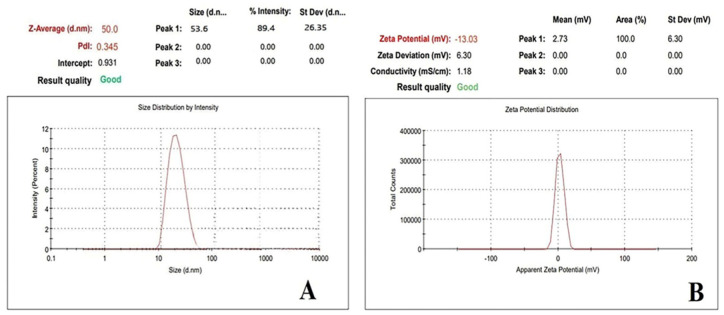
Depicts particle size (**A**) and zeta potential (**B**) for cytarabine-loaded TINPs (CY-TINPs).

Nanoparticle stability evaluation in the pH 7–8 range is a crucial criterion in assessing their utility in biomedical application. Thus, zeta potential measurements in water were used to investigate the aqueous stability of CY-TINPs and the efficacy of polymer coatings over nanoparticle surfaces. The pH of solid tumors, on average, ranges from 5.7 to 7.8 (with 7.0 being the common value), as has been demonstrated repeatedly in the scientific literature. While over 80% of pH values are all below 7.2 [42]. Extracellular pH is lower in malignant tumors compared to in healthy tissues [43]. When exposed to an acidic environment, nanoparticles develop a positive zeta potential and have a greater affinity for tumor tissues. Furthermore, the negative potential of the endothelial cell membranes in the tumor vasculature enhances the electrostatic interactions between the positively charged nanoparticles [44]. Thus, the positive charge of the TINPs and the negative charge of the cancerous cells or tissues account for the accumulation of nanoparticles at cancer sites and subsequently increased absorption of drug loaded TINPs in the targeted region. The zeta potential (−13.03 mV) and particle size (53.6 nm) obtained data depicted in Figure 5A,B respectively.

#### 3.1.5. Morphology of CY-TINPs Using TEM

**Figure 6:**
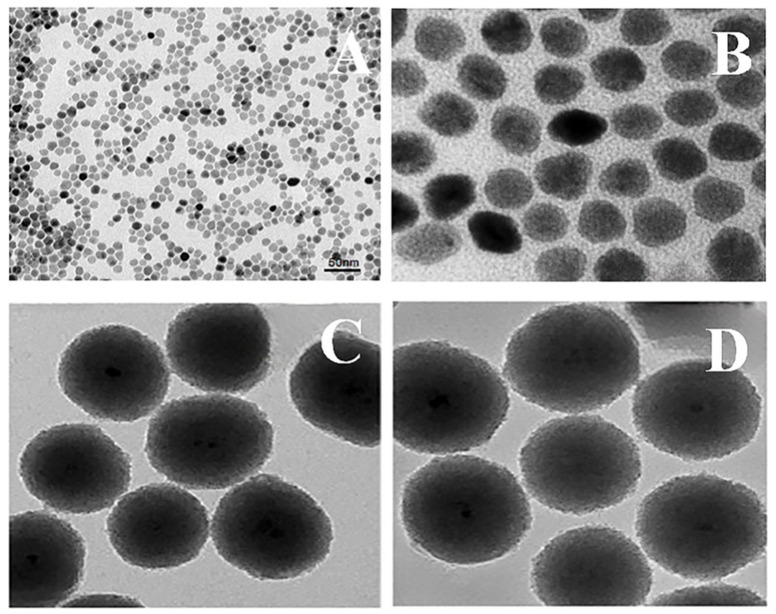
TEM morphological images for cytarabine-loaded TINPs (CY-TINPs) (**A**) after 3-month stability studies and (**B**–**D**) are ultra-magnified TEM morphological image.

TEM was used to study the particle morphology of cytarabine-loaded TINPs (CY-TINPs) and the images are exhibited in Figure 6. The CY-TINPs suggested uniform spherical transformation, and the particle size was found to be in the range of 50–60 nm. Reduced particle size helps the developed CY-TINPs to penetrate to the cellular level effectively and produce therapeutic response at the target site. The results of this imaging experiment are displayed in Figure 6A,B. The solid iron oxide core nanoparticle and coated polymeric covering system act like a lattice structure by providing tensile stability, regulated surface architecture, biodegradability with nano particle size distribution, and high surface area. The surrounding polymer shell is normally biocompatible and acts like a cell membrane. Van der Waals forces, hydrophobic interaction, electrostatic forces, hydrogen bonding, and other physical interactions all play a critical role in bonding polymeric covering materials and metallic cores in nanoparticles.

#### 3.1.6. Entrapment Efficiency (EE) and Drug Loading (DL) Parameter

To ascertain the drug load of the nanoparticles, ultracentrifugation was used, with minor adjustments made to account for the specific nature of CY-TINPs. However, ultracentrifugation was ineffective in removing the nanosized particles from the aqueous dispersion because of their elevated zeta potential at a pH of 6.0. Once the pH of their dispersion has been lowered to 1.2 with 0.1 M hydrochloric acid, CY-TINPs are easy to separate by centrifugation because they are small and have a low zeta potential. The EE and DL percentage along with other parameters are shown in Table 1.

#### 3.1.7. In Vitro Dissolution Studies

**Figure 7:**
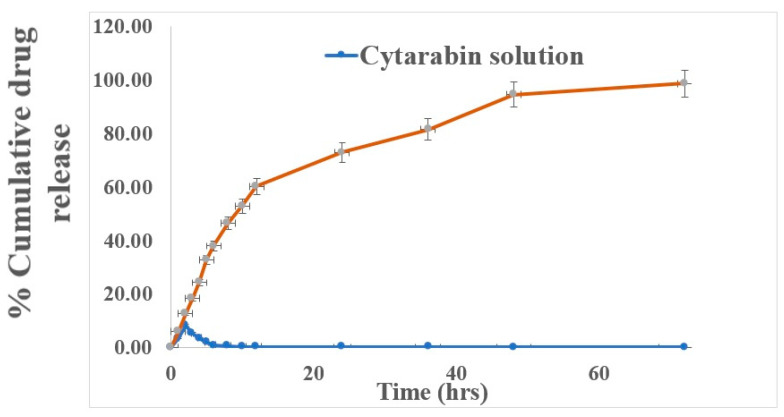
In vitro dissolution profile data for cytarabine solution and developed CY-TINPs formulation in PBS (PH 7.4).

Within 2 h, all the cytarabine had tendency to release in the dissolution medium. But it was sustained for up to 3 days from CY-TINPs when tested in vitro. Figure 7 depicts the cytarabine release profile from formulation. Instead of the typical burst release phenomenon, the nanoparticles gradually released the medication over a 72 h prolong time. The INPs nanostructure and trehalose surface coating is responsible for the drug’s slow release. In the beginning, formulation had a rapid release of the drug. The rapid release of medication adsorbed on the surface first and then the drug gets diffused slowly from the outer surface layer may be the possible mechanism. The reason behind this is that drug molecules on the nanoparticle surface rapidly dissolve itself due to cytarabine high aqueous solubility. After 2 h, the release rate slowed and remains sustained for the subsequent 72 h.

#### 3.1.8. Cell Viability Assay

**Figure 8:**
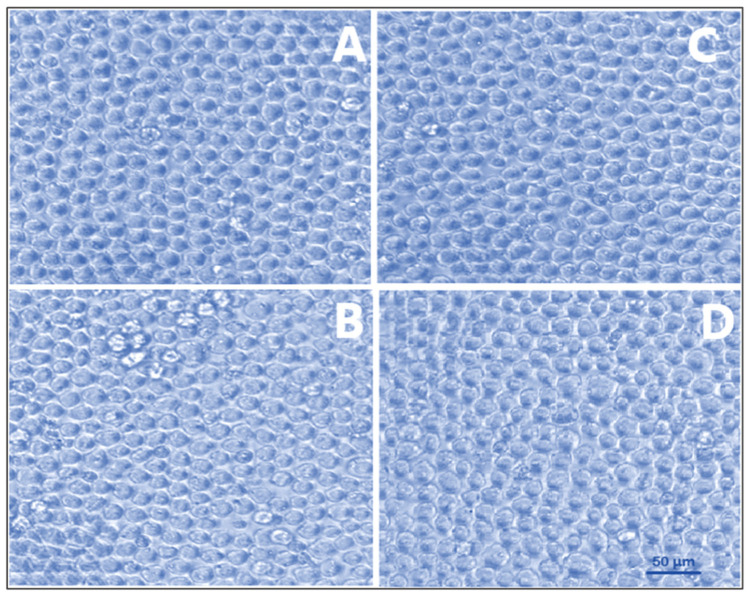
Morphological cellular appearance of human lymphocyte cells exposed for 72 h to materials and formulation such as (**A**) no treatment positive control, (**B**) only drug cytarabine, (**C**) neat TINPs, and (**D**) cytarabine-loaded TINPs (CY-TINPs).

**Figure 9:**
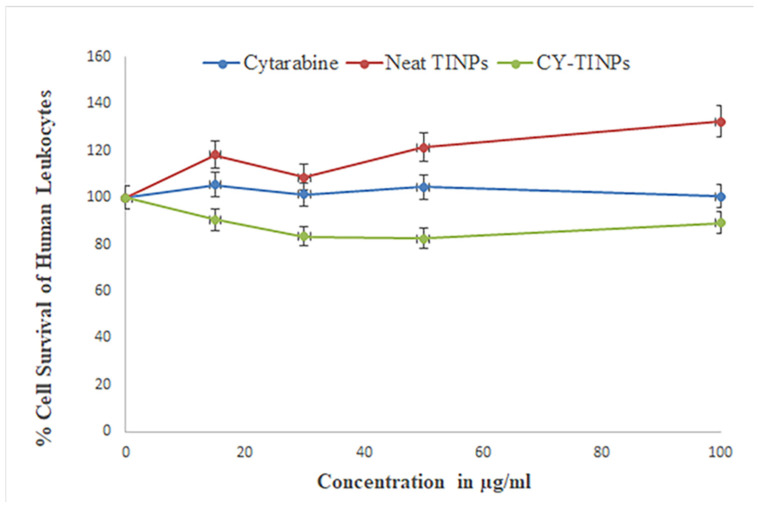
In vitro cell cytotoxicity study graph of cytarabine, neat TINPs, and cytarabine-loaded TINPs (CY-TINPs).

For therapeutic applications, the biocompatibility and safety of nanocarriers are of paramount importance. Therefore, it is important to search for nanomaterial that is both biocompatible and secure. The MTT assay was used to compare both unloaded and drug-loaded TINPs with normal human lymphocytes [29,45]. The results of the MTT assay demonstrated that normal human lymphocytes were not toxically affected by exposure to this CY-TINPs. As can be seen in Figure 8, the morphologies of normal and cytarabine-loaded TINPs lymphocytes did not significantly differ. At the highest dose examined (100 µg/mL), cell viability was greater than 82%. At the highest dose, cell viability was found to be 171 ± 1.8% with blank TINPs, 100 ± 0.6% with cytarabine, and 89 ± 2.3% with CY-TINPs (Figure 9). Therefore, these formulations were proven to be secure up to a concentration of 100 µg/mL. When the cell cycle is arrested in the S phase (DNA synthesis), cytarabine is rapidly transformed into its triphosphate form, which is toxic to DNA. Rapidly dividing cells are more vulnerable since DNA replication is necessary for mitosis. Long-term cytarabine use reduces cell proliferation and doubles the time. Our study also proved that cytarabine and CY-TINPs slow the rate at which cells can divide.

#### 3.1.9. Anticancer Activity

**Figure 10:**
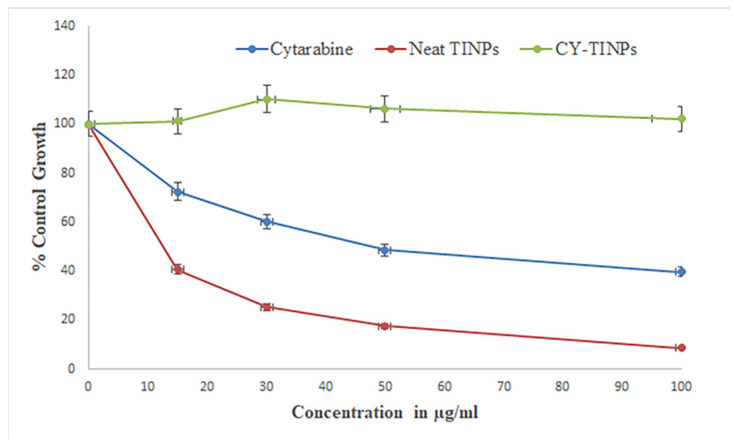
Anticancer activity graph of cytarabine, neat TINPs, and cytarabine-loaded TINPs (CY-TINPs).

**Figure 11:**
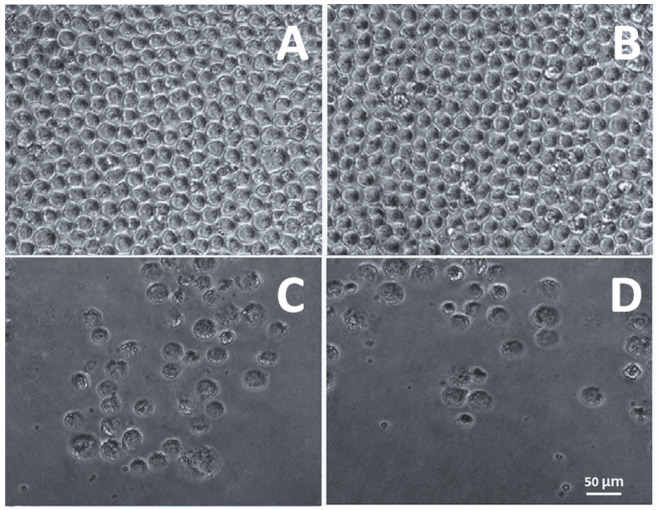
Morphological cellular appearance of HL 60 cells exposed for 72 h to materials and formulation such as (**A**) no treatment positive control, (**B**) only drug cytarabine, (**C**) neat TINPs, and (**D**) cytarabine-loaded TINPs.

HL-60 cells were used to assess the cytotoxicity of cytarabine-loaded TINPs by measuring their lymphocyte-killing activity [46]. Figure 10 depicts concentration-dependent percent growth control of TINPs and CY-TINPs on the LHL-600 cell line, implying concentration-dependent cell viability for both drug and formulation. TINPs without cytarabine was non-cytotoxic to HL60 cells; however, TINPs containing cytarabine (CY-TINPs) was more cytotoxic than cytarabine solution. This behavior may be attributed to cytarabine in TINPs, which boosted cellular uptake by being ingested by cells via an endocytosis process and then escaping from the endosomes and/or lysosomes into the cytoplasm. As shown in Figure 11, the number and shape of lymphocytes that had been treated with CY-TINPs were very different from lymphocytes that had not been treated.

## 4. In Vivo Studies of CY-TINPs Formulation

**Figure 12:**
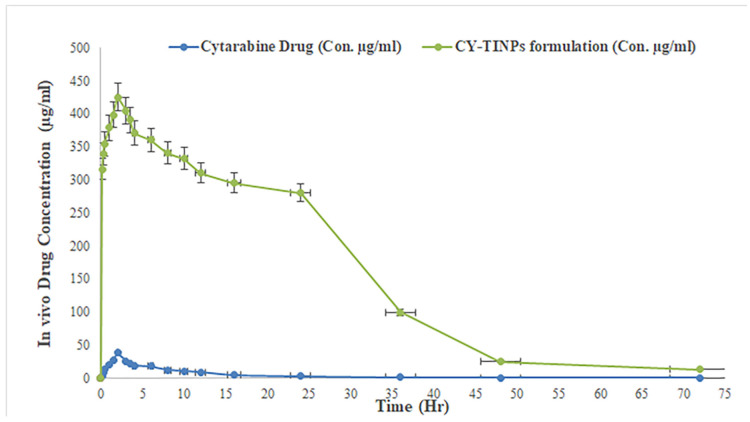
Depicts the serum concentration vs. time trends following single dose administration of cytarabine solution and CY-TINPs formulation.

The purpose of this study was to determine the efficacy of CY-TINPs in enhancing cytarabine bioavailability. Cytarabine was used as a drug in this study. Figure 12 depicts the drug plasma concentration vs. time trends following single dose administration of cytarabine solution and CY-TINPs formulation. Non-compartmental estimations of cytarabine PK parameters in rats for CY-TINPs formulation and cytarabine solution were performed using Kinetica 2000 software. The PK parameters AUCtotal, Tmax, Cmax, and t1/2 were computed and are displayed in Table 2. The student independent-samples test was used to compare the data statistically using GraphPad Prism software [47]. The plasma concentration value (Cmax) for CY-TINPs formulation (425.26 ± 2.11 µg/mL) was statistically significant with *p* value not >0.0001. While for cytarabine drug solution plasma concentration (Cmax) value was found to be 38.54 ± 1.03 µg/mL. The increased oral bioavailability of cytarabine from CY-TINPs could be attributed to a variety of causes. The retention of the drug at the site of absorption after oral delivery contributed significantly to the medication’s increased bioavailability [48]. Moreover, TINPs enhanced endocytic absorption and permeation by making the membrane more fluid; (ii) the formation of nano-sized structure greatly increases the effective surface area; (iii) long-chain trehalose improves drug absorption by reducing the rate at which the stomach empties; and (iv) fine-sized particles can move through the water-filled channels in the cell membrane. Furthermore, surface charge over CY-TINPs may have a substantial effect on cytarabine uptake across the GI membrane.

## 5. Conclusions

Cytarabine-loaded trehalose-coated iron oxide nanoparticles (Cy-TINPs) of size 50 nm were successfully synthesized and analyzed. The combined methodology of co-precipitation with impregnation of trehalose and drug loading was employed successfully to manufacture CY-TINPs with high entrapment efficiency (95%) and high drug loading capacity (28%). The XRD pattern and DSC data confirmed that cytarabine was entrapped within the carrier in stable form which was further displayed by the absence of drug peak in CY-TINPs. In vitro drug release studies of CY-TINPs indicated sustained release for a period of 72 hrs as compared to burst release of pure drug solution in PBS solution. Results from studies on human lymphocytes grown in vitro showed no discernible effect of the CY-TINPs formulation on cell survival. Cytarabine-loaded TINPs were shown to be more effective than cytarabine solution in inhibiting the growth of the HL60 cancer cell line. In vivo bioavailability of CY-TINPs was found to be enhance significantly as compared to drug solution. Developed formulation have successfully overcome the problems associated with cytarabine stability and instant degradation formation of inactive metabolites after administration. Developed CY-TINPs can be administered via intravenous route and have prolonged sustained release for 3 days with maintained concentration in the circulating blood of the patient. This formulation will benefit the cancer patients by providing them relief from higher dose toxicity problems.

## Figures and Tables

**Table 1:** Characterization and results of optimized cytarabine-loaded TINPs (CY-TINPs) (n = 5, mean ± SD).

Parameters	Results
Particle size	53.6 ± 1.65
Zeta potential	−13.03
Surface morphology by TEM	50 nm, uniform spherical shape
Entrapment efficiency	96.6 ± 1.74
Loading efficiency	28.35 ± 1.52
In vitro release profile	Sustained upto 72 h.
Cell viability studies	Nontoxic to Human cells
Anticancer activity	Improved cytotoxic potential in CY-TINPs than pure drug
Stability at 4–8 °C and room temperature	Stable at both temperature conditions

**Table 2:** Depicts the In vivo pharmacokinetic parameters for cytarabine drug and CY-TINPs formulation.

Parameters	Cytarabine Drug	CY-TINPs Formulation
C_max_ (ng/mL)	38.54 ±1.03	425.26 ± 2.11
T_max_ (h)	2.15 ± 1.15	2.55 ± 1.18
AUC_0–72_ (µg h/mL)	174.23 ± 58.18	11,546.64 ± 139.82
t_1/2_ (h)	1.0 ± 1.24	3.25 ± 1.33

## Data Availability

Not applicable.

## Abbreviations

INPs	Iron oxide nanoparticles
TINPs	Trehalose-coated stabilized iron oxide nanoparticles
CY-TINPs	Cytarabine-loaded trehalose-coated stabilized iron oxide nanoparticles
DSC	Differential scanning calorimetry,
XRD	X-ray powder diffraction
TEM	Transmission electron microscope
ROS	Reactive oxygen species
PEG-b-AGE	Polyethylene glycol-block-allyl anhydride ether
PEG	Polyethylene glycol
PCS	Photon correlation spectroscopy
PDI	Polydispersity index
TEM	Transmission electron microscopy
DL	Drug loading
EE	Entrapment efficiency
FBS	Fetal bovine serum
MTT	3-(4,5-Dimethylthiazol-2-yl)-2,5-diphenyltetrazolium bromide
DMSO	Dimethyl sulfoxide
CPCSEA	Committee for the Purpose of Control and Supervision of Experiments on Animals
FTIR	Fourier transform infrared
